# Anoikis related genes may be novel markers associated with prognosis for ovarian cancer

**DOI:** 10.1038/s41598-024-52117-0

**Published:** 2024-01-18

**Authors:** Chen Yang, LuChao Zhu, Qin Lin

**Affiliations:** Department of Radiation Oncology, Xiamen Cancer Center, Xiamen Key Laboratory of Radiation Oncology, The First Affiliated Hospital of Xiamen University, School of Medicine, Xiamen University, Xiamen, 361003 Fujian China

**Keywords:** Cancer genetics, Cancer genomics

## Abstract

The aim of this study was to determine the prognostic significance of anoikis related genes (ARGs) in ovarian cancer (OC) and to develop a prognostic signature based on ARG expression. We analyzed cohorts of OC patients and used nonnegative matrix factorization (NMF) for clustering. Single-sample gene-set enrichment analysis (ssGSEA) was employed to quantify immune infiltration. Survival analyses were performed using the Kaplan–Meier method, and differences in survival were determined using the log-rank test. The extent of anoikis modification was quantified using a risk score generated from ARG expression. The analysis of single-cell sequencing data was performed by the Tumor Immune Single Cell Hub (TISCH). Our analyses revealed two distinct patterns of anoikis modification. The risk score was used to evaluate the anoikis modification patterns in individual tumors. Three hub-genes were screened using the LASSO (Least Absolute Shrinkage and Selection Operator) method and patients were classified into different risk groups based on their individual score and the median score. The low-risk subtype was characterized by decreased expression of hub-genes and better overall survival. The risk score, along with patient age and gender, were considered to identify the prognostic signature, which was visualized using a nomogram. Our findings suggest that ARGs may play a novel role in the prognosis of OC. Based on ARG expression, we have developed a prognostic signature for OC that can aid in patient stratification and treatment decision-making. Further studies are needed to validate these results and to explore the underlying mechanisms.

## Introduction

Ovarian cancer (OC) is a common gynecologic malignancy that is highly prone to recurrence, metastasis, and drug resistance^1,2^. Despite the recent advancements in high-throughput sequencing technology and transcriptomic research, OC still lacks effective early tumor markers and diagnostic methods^3,4^. To address this issue, there is a pressing need to identify additional key driver genes, especially those that may impact the recruitment of the tumor microenvironment (TME) and immune infiltration in OC.

Anoikis is a process that occurs when cells detach from the correct extracellular matrix, disrupting integrin ligation and leading to cell death. This mechanism is crucial in preventing dysplastic cell growth and maintaining tissue homeostasis and development^5^. Anoikis related genes (ARGs) have been linked to a range of cancers, and their expression may serve as biomarkers for such diseases^6–9^. Several studies have shown that the onset of anoikis depends on both intrinsic and extrinsic pathways^10^. Multiple intracellular signals such as DNA damage and endoplasmic reticulum stress trigger apoptosis, and mitochondria play a central role in controlling anoikis^11^. This anoikis execution disorder may be a feature of cancer cells that contributes to tumor invasion and migration, the formation of distant organ metastases, and the development of drug resistance^12–14^. Anoikis is also correlated with the immune infiltration. Anoikis was found as an epigenetic driver of lymphocyte mimicry in aggressive cancers that links immune cell (IC) development to metastatic behavior^15^. What’s more, anoikis resistance may reshape the tumor microenvironment, resulting in immune evasion and induces chemoresistance^16,17^. However, previous studies exploring the relationship between OC and anoikis have only examined a limited number of genes^18–20^. Therefore, a comprehensive analysis of the association between OC and ARGs is necessary.

**Figure 1:**
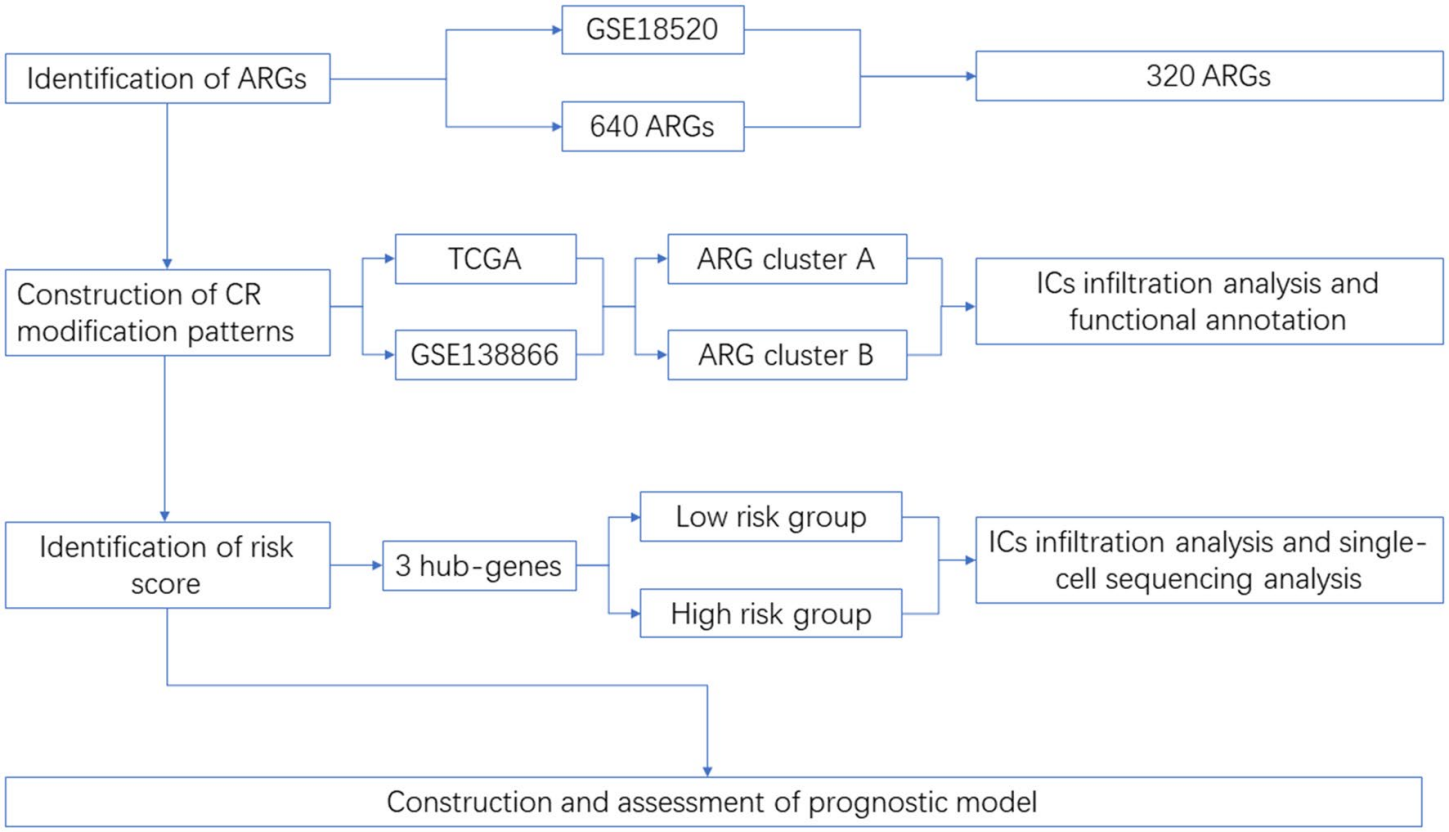
Flow chart of this study.

In this study, OC samples were collected from the Cancer Genome Atlas (TCGA) and the Gene-Expression Omnibus (GEO) to evaluate the role of anoikis in OC. The immune infiltration characteristics were evaluated by grouping patients into different patterns. A scoring system, constructed based on ARGs, was used to assess patients with different types and risk scores. The study's flowchart is illustrated in Fig. 1. The role of each dataset was shown in Table 1.

**Table 1:** Information about datasets.

ID	Dataset	Role	Histological sources
1	GSE18520	ARGs screening	Epithelium
2	TCGA-OV	Modal construction	Epithelium
3	GSE138866	Modal construction	Epithelium
4	GSE151214	Validation	Epithelium

*ARGs* anoikis related genes.

## Materials and methods

### Dataset acquisition and preprocessing

In order to identify differentially expressed anoikis related genes (DEARGs) between normal and OC tissues, the GSE18520^21^ dataset was analyzed using the “limma” package^22^. The criteria for DEARGs was set as |logFC| > 1 and adjusted P-value < 0.05. A total of 640 anoikis regulated genes (ARGs) were identified from Genecards with a relevance score > 0.2 and from Gilmore's study^23^. The genes we focused were the intersection of ARGs from GSE18520 and Genecards and 320 ARGs were eventually screened. The gene expression and corresponding clinical characteristics of 571 OC samples from the TCGA dataset and 126 OC samples from the GSE138866 dataset were integrated and analyzed, after controlling for batch effects using the “limma” package. Finally, after excluding samples without necessary survival and clinical labels, 697 OC patients were included in the study and the baseline characteristics was shown in Table 2.

**Table 2:** Baseline clinical characteristics of patients for model construction.

Characteristics	OC samples (n = 697)	
	TCGA-OV (n = 571)	GSE138866 (n = 126)
Age		
≤ 65	394	72
> 65	177	54
Grade		
G1	6	0
G2	69	0
G3	495	126
G4	1	0

*OC* ovarian cancer.

### Construction of anoikis modification patterns and functional annotation

The “Consensus Cluster Plus”^24^ package was used to identify different modification patterns of anoikis in OC patients using Nonnegative Matrix Factorization (NMF) clustering. To examine the differences in biological processes among different anoikis modification patterns, Gene Set Variation Analysis^25^ package was used to identify different modification patterns of anoikis in OC patients using Nonnegative Matrix Factorization (NMF) clustering. To examine the differences in biological processes among different anoikis modification patterns, Gene Set Variation Analysis^26^.

### Estimation of immune infiltration

To quantify IC infiltration into the TME of OC, we used single-sample gene-set enrichment analysis (ssGSEA) to estimate the immune infiltration between different anoikis modification patterns. The dataset for the types of ICs, containing 23 types, was obtained from Charoentong's research^27^. The CYBERSORTx tool was then used to calculate the correlation between the differentially expressed anoikis related genes (DEARGs) and ICs.

### Establishment of risk score and nomograms

The 697 OC patients were randomly divided into a training set and a testing set in a 1:1 ratio. The gene expression of DEARGs and survival labels were first obtained. Then, the LASSO algorithm was applied to the training set to screen for hub genes and reduce overfitting in the prognostic model. Finally, by performing multivariate Cox regression, the coefficients of the hub genes were determined and a risk score was calculated using the following formula.

$${\text{risk score}}={C}_{1}*{ARG}_{1}+{C}_{2}*{ARG}_{2}+{C}_{3}*{ARG}_{3}+\cdots +{C}_{n}*{ARG}_{n}$$

where C_i_ presented the coefficient of corresponding ARG and ARG_i_ presented the expression of certain ARG.

Patients were divided into two risk categories, low-risk and high-risk, based on their median score. To evaluate the effect of clinical features and risk scores on prognosis, multivariate analysis was performed for each risk group. The results were then represented and quantified using a nomogram, which calculated the probability of survival in patients with OC at 1, 3, and 5 years. The overall clinical benefit was determined using Decision Curve Analysis (DCA), which compared the net benefit provided by the risk scores and clinical characteristics.

### Validation of hub-genes by single-cell sequencing

A single-cell sequencing dataset (GSE151214^28^) consisting of 12 OC samples was used to confirm the expression of key genes in immune cells (ICs). The Tumor Immune Single Cell Hub (TISCH, http://tisch.comp-genomics.org)^29^ was utilized to perform various data quality checks, remove batch effects, cluster the cells, annotate cell types, classify malignant cells, and perform differential expression analysis.

### Statistical analysis

One-way ANOCA and Kruskal–Wallis tests were used to compare differences among groups. Subgroups were defined using the “survminer” package based on the relationship between risk factors^30^. Correlation between the expression of immune cells in the tumor microenvironment (TME) and ARGs was determined using Spearman and distance correlation analysis. The optimal parameters were identified by iteratively testing all potential cut points using the “surv-cutpoint” function. The Kaplan–Meier method was applied for prognostic analysis, and the significance was confirmed using log-rank tests. The prognostic analysis for OC patients was visualized using the “forestplot” package. The “timeROC”^31^ package was used to plot Receiver Operating Characteristic (ROC) curves and calculate the area under the curve (AUC). A significance level of P < 0.05 was used for all statistical tests. The analyses were performed and figures were generated using R 4.0.3 software^32^.

### Ethics approval and consent to participate

All data in our research was acquired from public datasets and did not require ethical review or informed consent. All humans were not directly involved in the study.

## Results

### Determination of DEARGs

**Figure 2:**
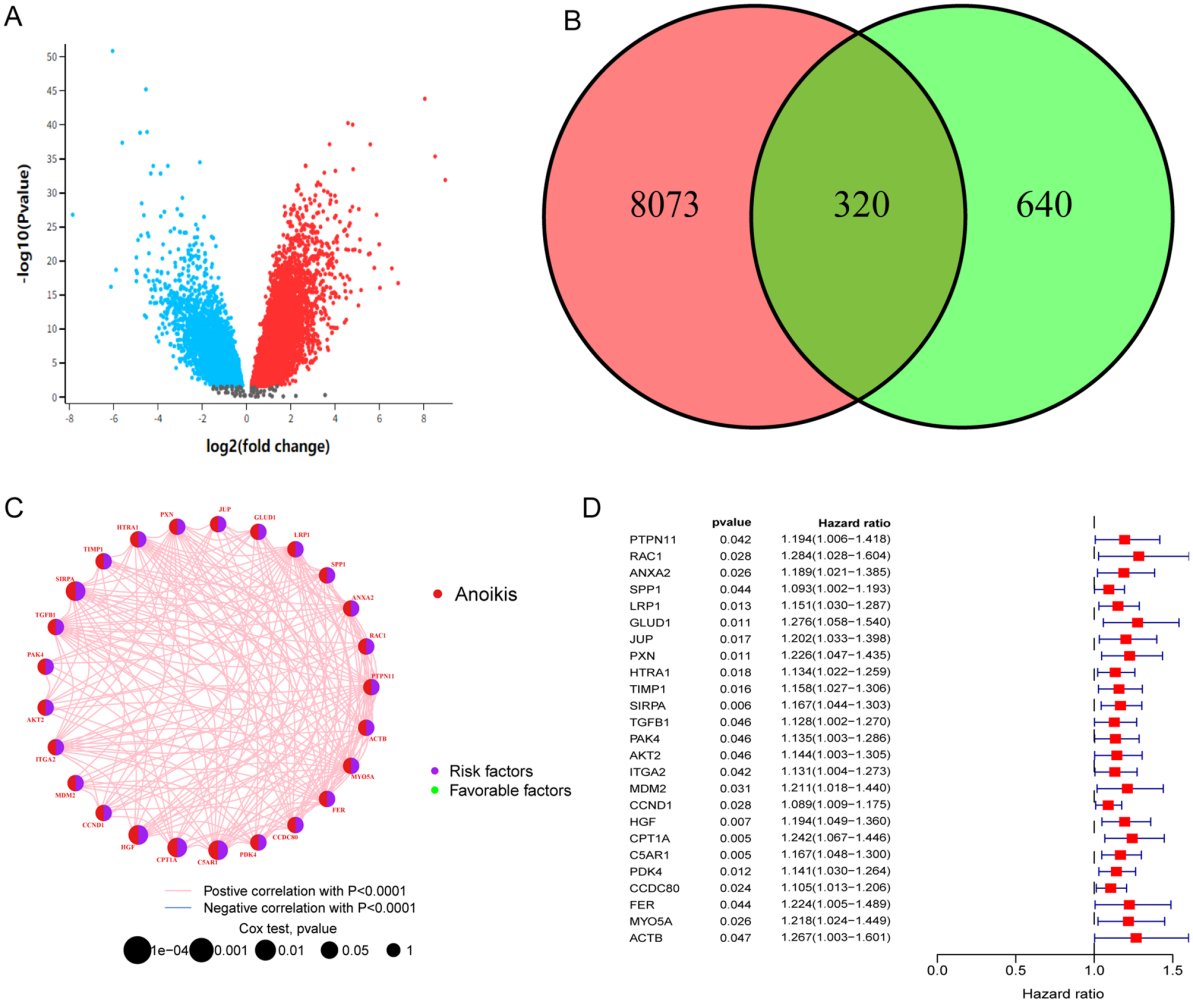
Landscape of ARGs in OC. (**A**) Visualization of gene expression in GSE18520. Blue dots represent genes with logFC descending greater than 1. Red dots represent genes with logFC ascending greater than 1. Black dots represent genes with logFC between − 1 and 1. (**B**) The Venn plot of intersection between .GSE18520 and anoikis gene set. Red for the number of DEGs identified from GSE18520. Green for the anoikis gene set. The number of intersected genes is 320. (**C**) The correlation between m6A regulators in OC. The lines linking regulators showed their interactions and red represent positive correlation while blue represents negative correlation. The erasers, readers and writers are colored red, orange, and grey, respectively. Green and purple dots in the circle represent protective and risk factors respectively the size of each circle represented the statistical P-value P < 0.0001, P < 0.001, P < 0.01, P < 0.05, respectively. (**D**) Visualization of impact of anoikis genes. The left part represents the gene symbol, P value and hazard ratio. The right part is the visualization of hazard ratio of anoikis genes. The dotted line represents that the hazard ratio equals 1. The boxes on the right of dotted line represents hazard ratio > 1 and on the right of dotted line represents hazard ratio < 1. The top and bottom of the boxes represents maximum to minimum values. Red lines in the boxes show the median value.

The GSE18520 dataset, which contained 10 normal samples and 53 OC samples, was analyzed, and 8,073 differentially expressed genes (DEGs) were identified using the criteria of |logFC| > 1 and adjusted P-value < 0.05 (Fig. 2A). By overlaying the anoikis gene set, 320 differentially expressed anoikis-related genes (DEARGs) were finally selected for further study (Fig. 2B). Univariate Cox regression analysis indicated that 25 DEARGs were significantly related to the overall survival (OS) of patients with OC (Fig. 2C), and the potential interactions among 31 DEARGs were revealed in Fig. 2D.

### Construction of anoikis modification patterns

**Figure 3:**
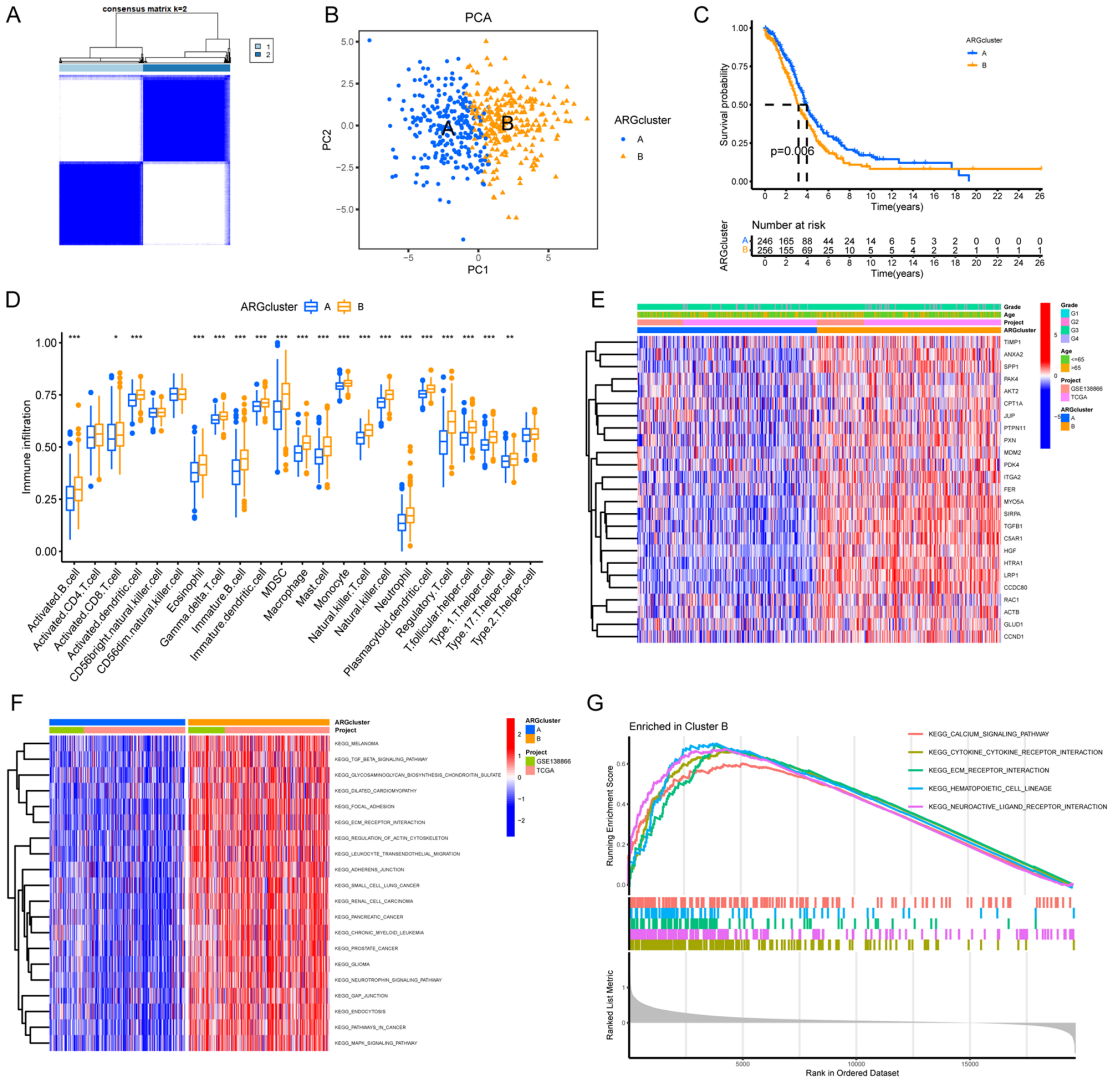
Construction of ARG clusters and characteristics of TME-infiltrated ICs. (**A**) Identification of OC modification patterns by nonnegative matrix factorization. (**B**) Transcriptome analysis of distinct ARG clusters with PCA. Blue for ARG cluster A and yellow for ARG cluster B. (**C**) Survival analyses of both ARG clusters. Blue for ARG cluster A and yellow for ARG cluster B. The number of alive patients along with time in both clusters is at the bottom of the picture. Kaplan–Meier curves show significant survival differences among both anoikis modification patterns, while arg cluster A exhibits a significant survival advantage. (**D**) ICs of TME infiltrating of distinct ARG clusters. Blue for ARG cluster A and yellow for ARG cluster B. The top and bottom of the boxes represents maximum to minimum values. Black dots represent outliers. Lines in the boxes show the median value. The asterisks represent the statistical P value (*P < 0.05; **P < 0.01; ***P < 0.001). (**E**) Visualization of patients’ characteristics and ARGs in distinct ARG clusters. In the heatmap, red represents increased expression of ARGs; blue represents decreased expression of ARGs. (**F**) Activation of biological pathways analysis in two ARG clusters with GSVA. The heatmap was a visualization of these biological processes. Red, activated pathways; blue, inhibited pathways. (**G**) Activation of biological pathways analysis in two ARG clusters with GSEA. ARG cluster B are found enriched in these pathways.

Based on the expression of the anoikis regulators, patients were divided into two distinct anoikis modification patterns using the “ConsensusClusterPlus”^24^ package, referred to as ARG cluster A and ARG cluster B, respectively (Fig. 3A). The OC samples could be separated into two clusters based on principal component analysis (PCA) (Fig. 3B). The survival analysis revealed that ARG cluster A showed a significantly better survival outcome compared to ARG cluster B (Fig. 3C). Further analysis of both ARG clusters and the clinical features of patients was conducted using a heatmap, which showed that distinct immune cell (IC) clusters had a significant difference in transcriptional profiles (Fig. 3E). As ARG cluster A was characterized by decreased expression of ARGs, this suggested that high expression of ARGs might be a risk factor for patients with OC. The investigation of the innate IC infiltration in the tumor microenvironment (TME) (Fig. 3D) also showed significant differences in infiltration levels, including Activated B Cells, Activated Dendritic Cells, Eosinophils, Immature B Cells, Immature Dendritic Cells, and myeloid-derived suppressor cells (MDSCs) among the two ARG clusters. Most ICs were found to be universally enriched, indicating that IC enrichment in OC may be associated with a poor prognosis. Gene set enrichment analysis (GSEA) and gene set variation analysis (GSVA) were conducted to investigate the biological behavior of the two ARG clusters. As shown in Fig. 3F,G, both ARG clusters showed differences in the enrichment of biological pathways.

### Biological features of risk score

**Figure 4:**
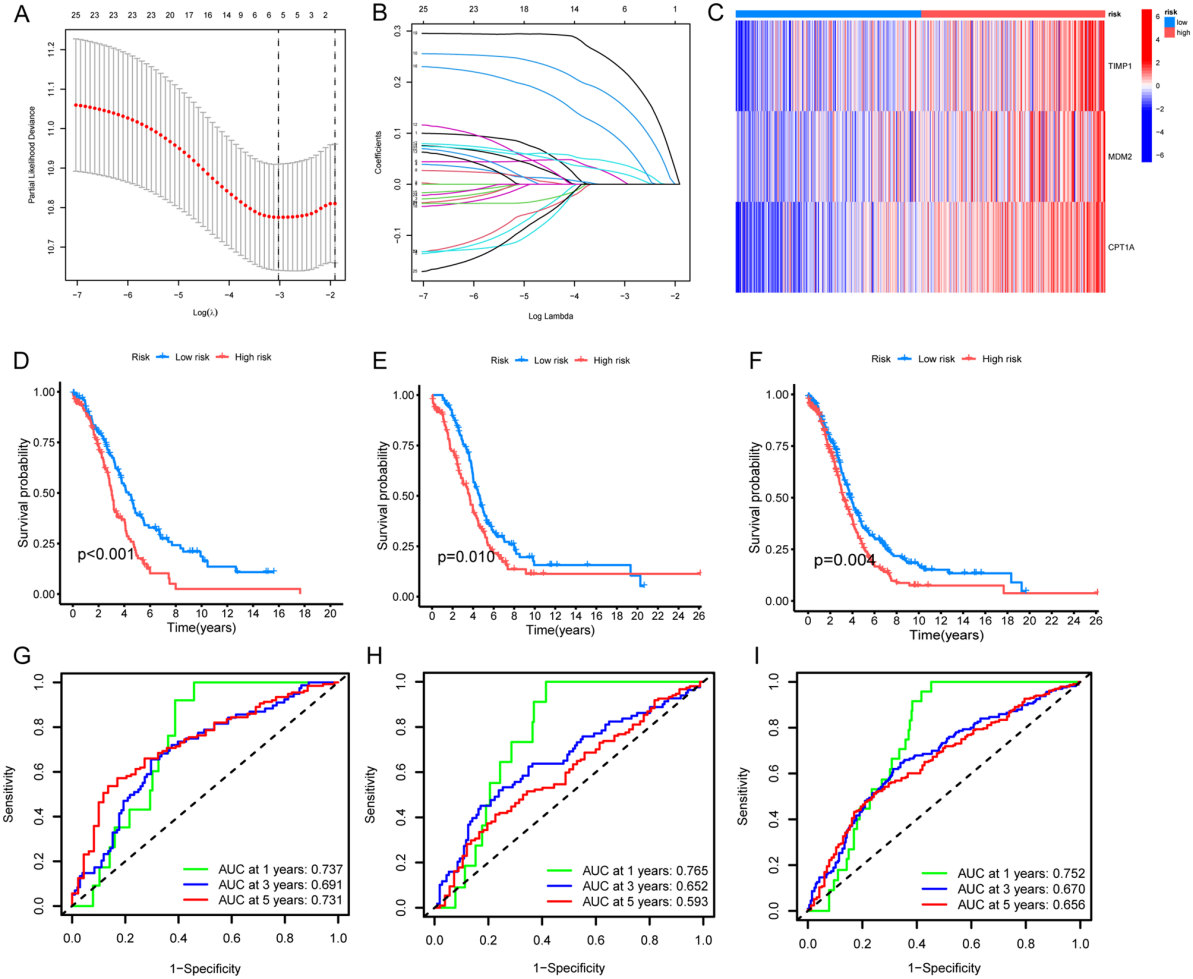
Construction of anoikis phenotype-related genes signatures. (**A**,**B**) Seek for optimal regularization coefficient of LASSO via Gradient descent. (**C**) Visualization of the expression of hub-genes in both risk groups. Red represents increased expression. Blue represents decreased expression. (**D**–**F**) Survival analyses of different risk groups in training cohort, testing cohort and all-patients cohort. Blue for low risk group and red for high risk group. The number of alive patients along with time in three clusters was at the bottom of the picture. Kaplan–Meier curves showed significant survival differences among the three ARG modification patterns, while ARG cluster A exhibits a significant survival advantage. (**G**) Training cohort; (**H**) Testing cohort; (**I**) All-patients cohort. (**G**–**I**) ROC curve of ARG modes for 1 year, 3 years and 5 years in training, testing and all-patients cohort. Green for 1 year, blue for 3 years and red for 5 years. (**C**) Training cohort; (**D**) Testing cohort. E.All-patients cohort.

The LASSO algorithm was employed to analyze ARG modification patterns based on differentially expressed genes (DEGs) and to construct a risk score (see Fig. 4A,B). Additionally, the LASSO algorithm was utilized to assess ARG modification patterns based on DEGs and derive a risk score (Fig. 4C,E). Subsequently, a survival analysis was conducted on the training, testing, and entire patient cohorts, revealing a higher survival rate among patients in the low-risk group (see Fig. 4D–F).

To further evaluate the performance of the hub genes, Receiver Operating Characteristic (ROC) curves and Area Under the Curve (AUC) values were utilized. The results demonstrated strong predictive capabilities for survival at 1, 3, and 5 years across all three patient groups (see Fig. 4G–I).

### Immune characteristics of risk score

**Figure 5:**
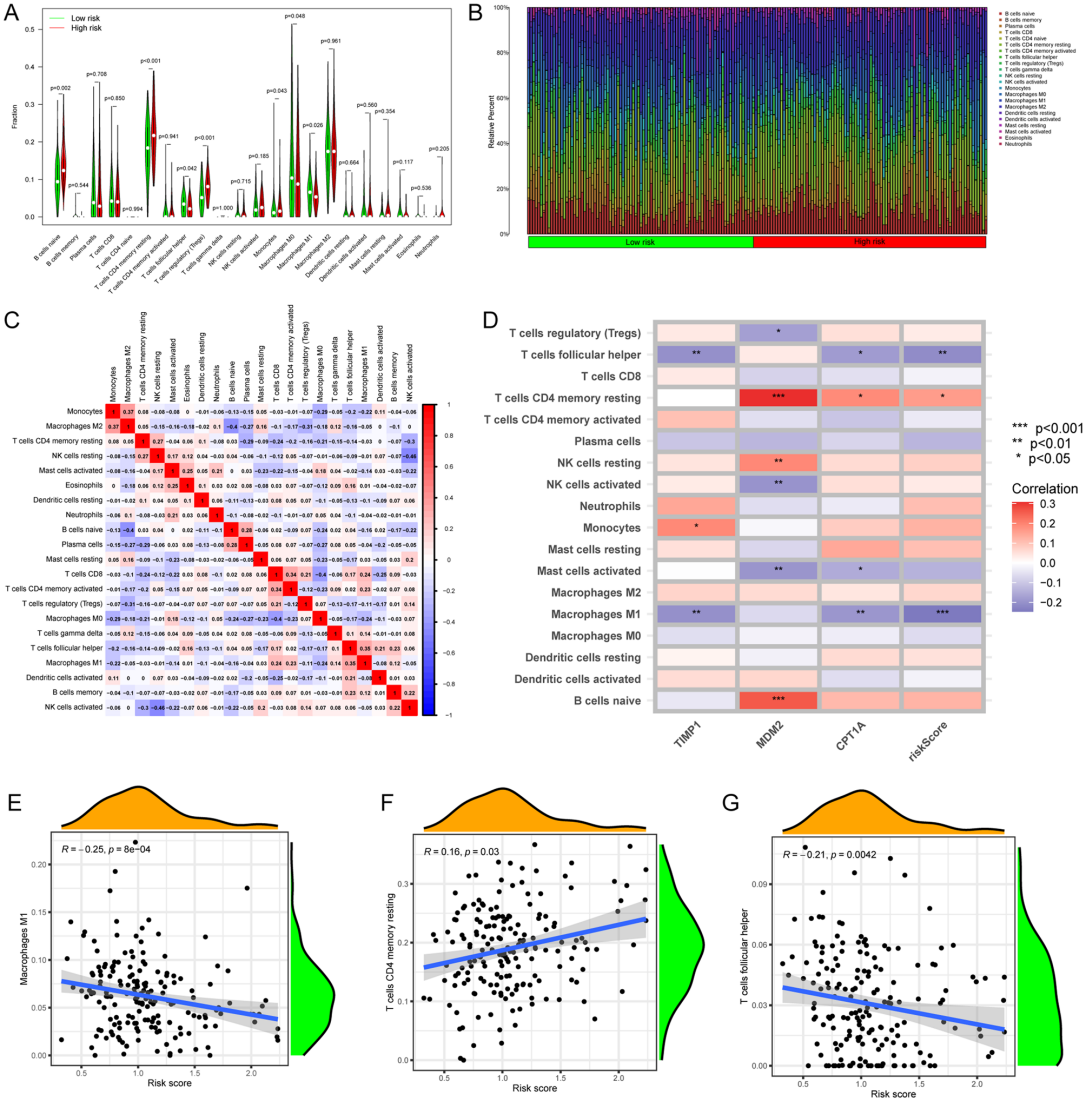
Construction of risk score. (**A**) ICs of TME infiltrating of distinct risk groups. Green for low risk group and yellow high risk group. The top and bottom of the boxes represents maximum to minimum values. Dots in the boxes show the median value. (**B**) Visualization cells infiltration in each OC samples. Each column represents an OC sample. The length of each box in each single sample represents the proportion of infiltration of the cell. (**C**) The correlation between ICs. Blue for negative correlations and Red for positive correlations of the two corresponding ICs. (**D**) The correlation hub-genes, risk score and ICs. Each row represents one IC. The columns are three hub-genes and risk score from left to right. (**E**–**G**) The relevance between risk scores and ICs.

To explore the association between hub genes and immune cell (IC) infiltration, we applied the CIBERSORTx algorithm to assess the proportion of immune cells. Additionally, we conducted an in-depth analysis of the correlation between immune cells and hub genes, as illustrated in Fig. 5A–D. Remarkably, we observed a noteworthy positive correlation between the risk score and CD4 memory resting T cells, underscoring a potential link between risk assessment and the presence of this specific T cell population. Conversely, a negative correlation was identified with M1 macrophages and follicular helper T cells, suggesting a potential immunological mechanism underlying the risk score (Fig. 5E–G).

### Validation of hub-genes by single-cell sequencing

**Figure 6:**
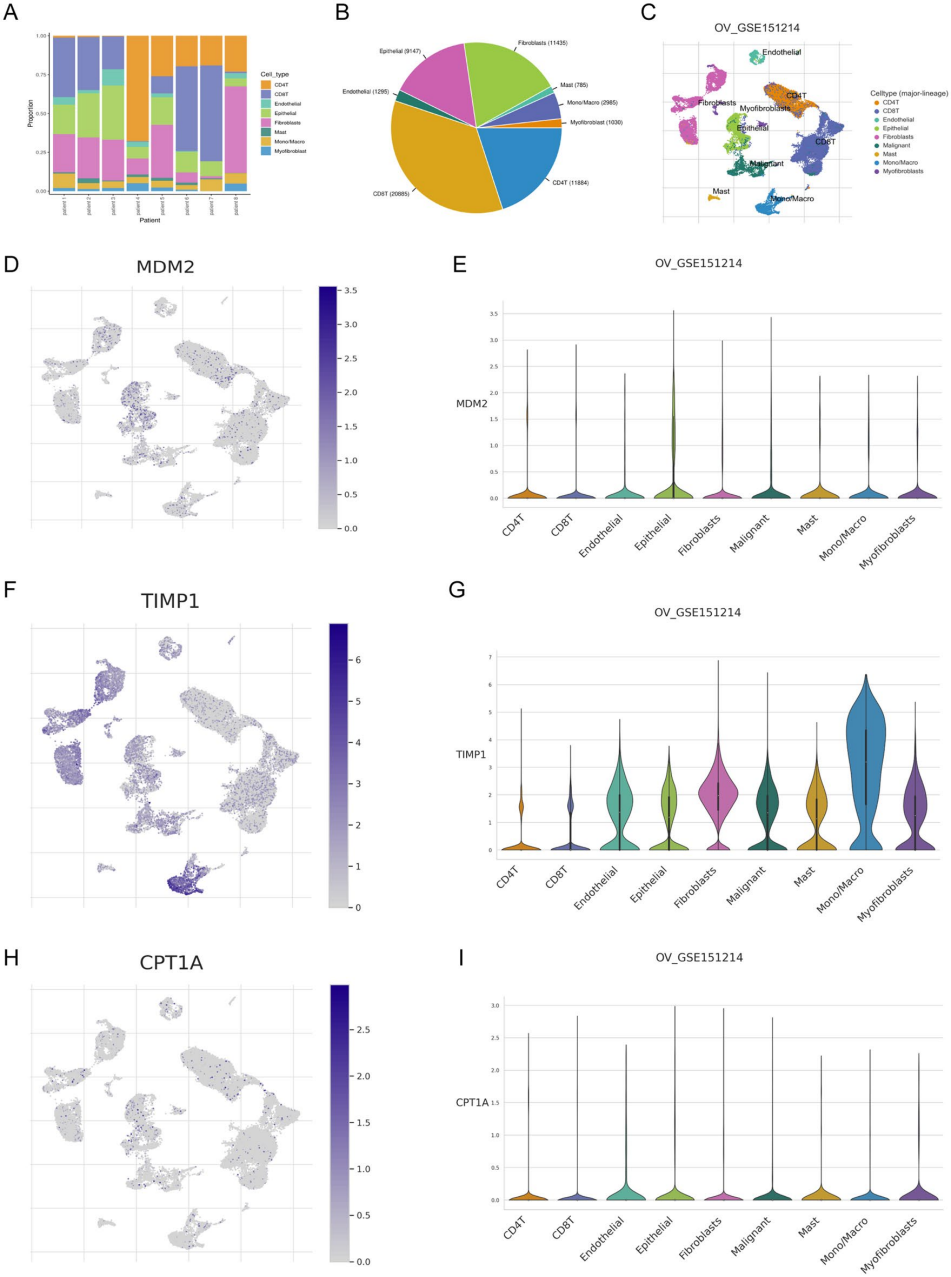
Establishment of prognosis model. (**A**) The distribution of cells in each sample in GSE151214. Each column represents a single OC case. The ordinate is the proportion of cells. The box in each column represents the proportion of this cell. (**B**) Visualization of percentage of absolute cell number in all OC cases. (**C**) Clusters and cell annotation for OC cases. (**D**) The expression of *MDM2* in cells. The background represents cell and blue dots represent *MDM2* expressed in this cell. (**E**) The relative expression of *MDM2* in each kind of cells. The abscissa is each cell and the coordinate is the relative expression quantity. (**F**) The expression of *TIMP1* in cells. The background represents cell and blue dots represent *TIMP1* expressed in this cell. (**G**) The relative expression of *TIMP1* in each kind of cells. The abscissa is each cell and the coordinate is the relative expression quantity. (**H**) The relative expression of *CPTA1* in each kind of cells. The abscissa is each cell and the coordinate is the relative expression quantity. (**I**) The relative expression of *CPT1A* in each kind of cells. The abscissa is each cell and the coordinate is the relative expression quantity.

To meticulously validate the expression profiles of the hub genes, we conducted a rigorous analysis employing a single-cell sequencing dataset (GSE151214). Subsequently, this dataset underwent a comprehensive assessment utilizing TISCH, a robust tool adept at batch effect correction, clustering, and precise cell-type annotation, as illustrated in Fig. 6A–C. Notably, *MDM2* exhibited the highest abundance within the epithelial cell group, while *TIMP1* demonstrated prominent prevalence within the mono/macro cell group. Additionally, *CPT1A* emerged as the most abundantly expressed gene in myofibroblasts.

The detailed evaluation of the relative abundance of hub genes across diverse cell types is visually presented in Fig. 6D–I. These findings provide compelling evidence indicating that the hub genes may wield a substantial influence on the composition and dynamics of the TME.

### Establishment of prognostic signature

**Figure 7:**
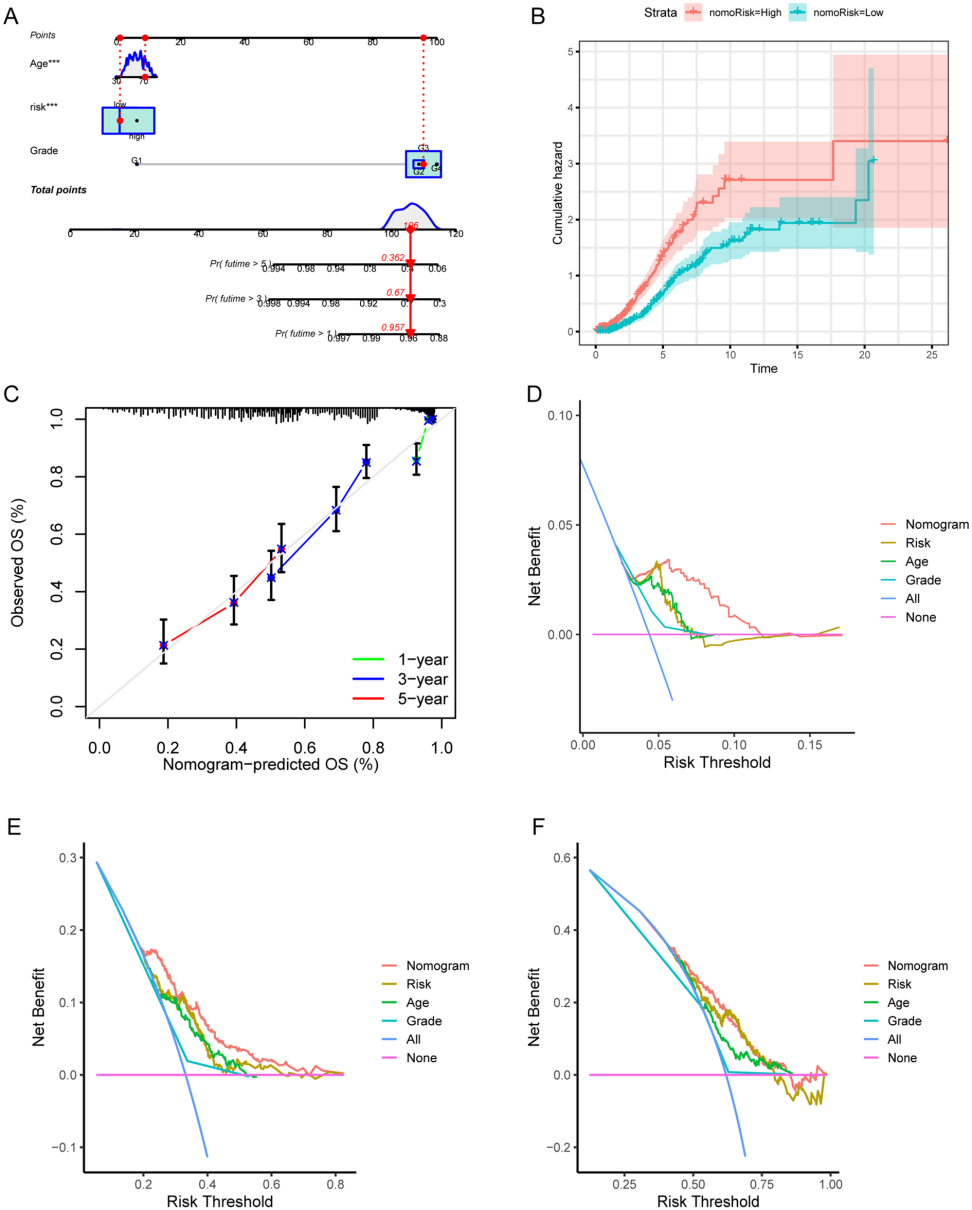
Immune characteristics of risk score. (**A**) Visualization of multivariate analysis for age, grade, and risk. Significant correlation is found between prognosis with age and risk score. (**B**) Cumulative analysis of nomogram. Red for high risk group and green for low risk group. The abscissa represents the survival time and the ordinate represents the Cumulative hazard. (**C**) The calibration curves for overall survival in 1 year, 3 years and 5 years. Green for 1 year, blue for 3 years and red for 5 years. (**D**–**F**) Clinical decision curve for prognostic model in training cohort, testing cohort and all-patients cohort. Orange represents the nomogram. Purple represents risk. Green represents age. Wathet represents grade. Dark blue represents all factors. Pink line represents model without any genes.

A comprehensive multivariate analysis was conducted to assess the impact of clinical features, including grade, age, and risk score, on the prognosis of OC. The survival outcomes of OC patients were vividly presented and quantified using a nomogram plot, as exemplified in Fig. 7A. The cumulative score from each covariate provides a valuable tool for predicting individual patient survival rates at 1, 3, and 5 years. Furthermore, the cumulative hazard analysis, illustrated in Fig. 7B, demonstrated the effectiveness of the risk stratification approach. Additionally, the calibration curve in Fig. 7C showcased that the signature's performance was well-controlled to prevent overfitting. In addition, the decision curve analyses (DCAs) at 1 year (Fig. 7D), 3 years (Fig. 7E), and 5 years (Fig. 7F) underscored that the nomogram yielded a substantially higher net clinical benefit, further affirming its utility as a prognostic tool.

## Discussion

The role of anoikis in regulating the biological behavior and heterogeneity of tumor cells has been well established^33^. For example, *IQGAP1* has been reported to activate the Src/FAK pathway, enhancing cell viability and inhibiting anoikis in hepatocellular carcinoma, making it a valuable indicator of metastasis and prognosis^34^. Additionally, the activation of *CPT1A *has been shown to facilitate metastasis and confer anoikis resistance in colorectal cancer^35^. Furthermore, *CCN2* has been demonstrated to block lung cancer development by inhibiting the anoikis pathway related to DAPK^36^. Thus, investigating the prognostic value of ARGs may lead to the discovery of new treatment targets and markers for multiple cancers.

In our study, we identified three hub genes, *TIMP1*, *MDM2*, and *CPT1A*, as potential risk factors for OC. Our findings suggest that the increased expression of these hub genes is associated with an increased risk of OC.

*TIMP1 *(Tissue Inhibitor of Metalloproteinases 1), which is overexpressed in OC, has been shown to affect the tumor microenvironment (TME) by altering the behavior of both tumor and endothelial cells, leading to drug resistance, particularly in advanced OC patients^37^. *TIMP1* is a protein that belongs to a family of proteins known as tissue inhibitors of metalloproteinases. These proteins play a crucial role in regulating the activity of matrix metalloproteinases (MMPs), which are enzymes responsible for degrading the extracellular matrix. The extracellular matrix is a complex network of proteins and carbohydrates that provides structural support to cells and helps in various cellular processes. Studies have shown that *TIMP1* expression can be altered, and its dysregulation has been associated with the progression and metastasis of OC^38^. Elevated levels of *TIMP1* have been observed in OC tissues and in the serum of OC patients. This suggests that *TIMP1* may play a role in tumor invasion, angiogenesis, and metastasis in OC. Additionally, *TIMP1* in combination with VEGF has been linked to tissue invasion and angiogenesis in OC^39^.

*MDM2* (Mouse Double Minute 2 homolog), on the other hand, has been shown to be a critical regulator of OC metastasis. *MDM2* is a critical cellular protein that plays a central role in regulating the activity of the tumor suppressor protein p53^40^. *MDM2* primarily functions as an E3 ubiquitin ligase, targeting p53 for degradation by the proteasome. This interaction is part of a negative feedback loop that helps to tightly control p53 levels in normal cellular processes. *MDM2* has been found to be overexpressed in some cases. Elevated levels of *MDM2* can lead to decreased p53 activity, as *MDM2* promotes the degradation of p53, thereby reducing its tumor-suppressive functions. This dysregulation of the p53 pathway can contribute to uncontrolled cell growth and the development or progression of cancer. Chen demonstrated that *MDM2* activated the Smad pathway and drives OC metastasis. Targeting the N-terminal of *MDM2* has the potential to re-program epithelial-mesenchymal transition (EMT) and prevent cancer cells from migrating^41^.

*CPT1A* (Carnitine Palmitoyltransferase 1A) is an enzyme that plays a crucial role in fatty acid metabolism. It is responsible for the transport of long-chain fatty acids into the mitochondria, where they can be oxidized to generate energy. This process is essential for providing energy to cells, particularly in situations where glucose availability is limited. Research has shown that *CPT1A* expression may be altered. Some studies suggest that elevated levels of *CPT1A* may be associated with more aggressive forms of OC^42^. This is thought to be related to the increased demand for energy and biosynthetic building blocks that cancer cells require for their rapid growth and proliferation. Additionally, *CPT1A* may play a role in modulating cellular responses to stress and nutrient availability. *CPT1A* has been identified as a potential therapeutic target in OC through multiomic analysis. This is believed to be due to its ability to regulate cell cycle progression by repressing FoxO transcription factors in OC, as demonstrated by Shao^43^. These findings highlight the potential value of *CPT1A* as a prognostic marker for OC, making it a promising target for further investigation.

This study has several limitations that should be acknowledged. First, the origins of OC are diverse and we merely included epithelial originated OC samples in the study which means it is hard to be positive when applying the model on other types of OC. Second, only age and grade were included as clinical characteristics factors in constructing the prognostic model due to a lack of available information. What’s more, we did not conduct functional experiments in vitro or in vivo, which limits our understanding of the precise mechanism of action of these hub genes. Additionally, the sample size used in our study was relatively small, which may impact the robustness and stability of our model. It is also important to note that this study was retrospective in nature and did not utilize prospective data to test the model's performance. As a result, it is essential to validate the signatures identified in this study with larger, prospective datasets in order to increase their precision and reliability. Future studies could also explore the specific characteristics and genetic features of those subtypes, which would help build a more comprehensive understanding of the heterogeneity within OC as a whole to extend the applicability to different types of OC.

## Conclusions

Our study aimed to examine the effects of anoikis modification on the regulation mechanisms of OC. Our findings suggest that different patterns of anoikis modification can result in heterogeneity within the tumor microenvironment (TME), creating varying outcomes in OC progression. Through the exploration of these patterns, we aim to enhance our understanding of the role of the TME and immune infiltration in OC. This, in turn, could lead to the development of more personalized treatment approaches for patients with OC.

## Data Availability

All data in our research can be acquired from TCGA datasets (https://portal.gdc.cancer.gov/) and GEO datasets (https://www.ncbi.nlm.nih.gov/geo/query/acc.cgi?acc=GSE18520, https://www.ncbi.nlm.nih.gov/geo/query/acc.cgi?acc=GSE138866 and https://www.ncbi.nlm.nih.gov/geo/query/acc.cgi?acc=GSE151214). All figures were generated by an open source software R.
